# Neutralization diversity of HIV-1 Indian subtype C envelopes obtained from cross sectional and followed up individuals against broadly neutralizing monoclonal antibodies having distinct gp120 specificities

**DOI:** 10.1186/s12977-021-00556-2

**Published:** 2021-05-14

**Authors:** Ranajoy Mullick, Jyoti Sutar, Nitin Hingankar, Suprit Deshpande, Madhuri Thakar, Seema Sahay, Rajesh P. Ringe, Sampurna Mukhopadhyay, Ajit Patil, Shubhangi Bichare, Kailapuri G. Murugavel, Aylur K. Srikrishnan, Rajat Goyal, Devin Sok, Jayanta Bhattacharya

**Affiliations:** 1 HIV Vaccine Translational Research Laboratory, Translational Health Sciences & Technology Institute, Faridabad, Haryana India; 2 International AIDS Vaccine Initiative, New Delhi, India; 3 ICMR-National AIDS Research Institute, Pune, Maharashtra India; 4 CSIR-Institute of Microbial Technology, Chandigarh, India; 5 Y R Gaitonde Center for AIDS Research & Education, Chennai, India; 6 IAVI Neutralizing Antibody Center, The Scripps Research Institute, La Jolla, CA USA; 7 Mississauga, ON L5B3Y9 Canada

**Keywords:** HIV-1, Clade C, Neutralizing antibodies, Envelope, VRC01, CAP256-VRC26.25, PGDM1400, PGT121, India

## Abstract

**Background:**

The potential use of the broadly neutralizing monoclonal antibodies (bnAbs) towards prophylaxis and treatment to HIV-1 is currently being explored. While a number of promising bnAbs have been discovered and a few of them have progressed towards clinical development, their extent of neutralization coverage with respect to global HIV-1 variants given the existence of genetically distinct subtypes and recombinants circulating globally is not clearly known. In the present study, we examined the variation in the neutralization susceptibility of pseudoviruses expressing 71 full length primary HIV-1 subtype C *envs* obtained from limited cross-sectional individuals over different time points against four bnAbs that target gp120 with distinct specificities: VRC01, CAP256-VRC26.25, PGDM1400 and PGT121.

**Results:**

We found significant variations in the susceptibility of Indian clade C to these four bnAbs. These variations were found to be distinct to that observed in African subtype C based on the existing datasets and concordant with their sequence diversity. Trend analysis indicated an increasing neutralization resistance observed over time with CAP25-VRC26.25, PGDM1400 and PGT121 when tested on pseudoviruses expressing *envs* obtained from 1999 to 2016. However, inconsistent trend in neutralization susceptibility was observed, when pseudoviruses expressing *envs* obtained from three followed up individuals were examined. Finally, through predictive analysis of the 98 Indian subtype C including those assessed in the present study by employing additive model implemented in CombiNAber (http://www.hiv.lanl.gov), we observed two possibilities where combinations of three bnAbs (VRC01/CAP56-VRC26.25/PGT121 and PGDM1400/CAP256-VRC26.25/PGT121) could achieve near 100% neutralization coverage.

**Conclusions:**

Our findings not only indicate disparate intra-clade C genetic vis-à-vis neutralization diversities but also warrant the need for more comprehensive study using additional isolates towards comparing inter and intra-clade neutralization diversities which will be necessary for selecting the bnAb combinations suitable for optimal coverage of the region-specific HIV-1 circulating subtypes. Expanding these efforts is imperative for designing efficacious bnAb based intervention strategies for India as well as subtype C in general.

**Supplementary Information:**

The online version contains supplementary material available at 10.1186/s12977-021-00556-2.

## Introduction

The development of a preventive vaccine for HIV-1 with a potential to tackle the enormous genetic diversity remains a challenge. This genetic variation is primarily accounted for by the HIV-1 *envelope* gene *(env)* which is responsible for greater than 35% difference in amino acid sequences between different subtypes [1]. A number of broadly neutralizing monoclonal antibodies (bnAbs) capable of cross neutralizing diverse genetic subtypes have been discovered from elite neutralizers since 2009, which have fuelled in recent times their potential use in prevention and therapy over and above the existing antiretroviral (ARV) drugs [2–9]. Few of these bnAbs have also advanced into clinical development based on their promising antiviral activity observed in animals and human clinical trials [10–16]. In general, breadth and potency of bnAbs are defined by their extent in neutralizing a panel of pseudoviruses expressing *envs* derived from various subtypes and recombinants representing different geographic regions. Hence, the suitability of bnAbs that exhibited the highest breadth to overcome region-specific diversity is not clearly known. Previous studies have shown that although bnAbs isolated from individuals infected with one particular subtype are generally effective at neutralizing viruses belonging to other subtypes, antibody potency is often found to be correlated with matched subtypes [17–21]. Moreover, viral and host diversities have been found to have an impact even within a matched subtype, as demonstrated by the fact that the subtype-matched neutralization advantage was more apparent in regions with distinct viral diversities [19, 22, 23].

HIV-1 subtype C accounts for approximately half of the global infections [24], which predominates in India and South Africa. Recently very few studies have attempted to understand a few selected bnAbs for their extent to neutralize HIV-1 subtype C of African origin either singly and/or in combinations [25–27], however, those studies did not include pseudoviruses expressing *envs* representing Indian subtype C. It is possible that an intra-clade C specific neutralization patterns may exist. For example, Rademeyer et al. [28] have shown that subtype C viruses of African origin have become more resistant to VRC01, PG9 and 4E10 compared to CAP256-VRC26.25 and PGT128. In the present study, we examined the variation in neutralization sensitivity of pseudoviruses expressing 71 complete HIV-1 subtype C primary *envs* collected over a period of time (1999–2014) against four bNAbs (VRC01, CAP256-VRC26-25, PGDM1400 and PGT121) that are not only the broadest and most potent amongst the best bnAbs discovered with distinct epitope specificities on viral envelope to date but also amongst ones that are farthest in under clinical development and testing [29–31].

## Results

### Evidence of variation in susceptibilities of HIV-1 India clade C to bnAbs targeting gp120 having distinct target epitope specificities

We first examined the degree of neutralization sensitivity of HIV-1 Indian clade C to four bnAbs: VRC01 (having CD4-binding site specificity), PGDM1400 and CAP256-VRC26.25 (both having V1V2 specificity) and PGT121 (target V3 supersite). A total of 71 pseudoviruses expressing complete *env* gene (*gp160)* obtained from 40 disease stage specific samples from 28 HIV-1 infected individuals from Southern and Western India during the years 1999–2014 (the complete details of these *env* clones along with their genetic properties are given in Additional file 1: Table S1) were assessed for their degree of neutralization sensitivity towards four selected bNAbs of distinct specificities in TZM-bl neutralization assay. Several of these *envs* have been reported earlier [32–38]. As shown in Table 1, the degree of susceptibility of Env-pseudotyped viruses to the bnAbs varied. The neutralization data demonstrated that while VRC01 exhibited most breadth (neutralized 62/71 viruses), PGDM1400 exhibited least (46/71) of all the four bnAbs assessed. The neutralization coverage was assessed by measuring the ability of each of the bnAb to demonstrate 50% virus neutralization by IgG concentration less than or equal to 5 µg/ml (IC_50_). In addition, CAP256-VRC26.25 was found to neutralize the virus panel with maximal potency (IC_50_ of 0.0033 with 76% breadth). Interestingly, while neutralization resistance of Env-pseudotyped viruses to all four bnAbs was found to be associated with known resistance signatures (Additional file 2: Table S2), for a few *envs,* no known resistance signatures were evident, suggesting an alternate mechanism of neutralization resistance. Overall, our data showed variation in neutralization breadth and potency of VRC01, CAP256-VRC26.25, PGDM1400 and PGT121 to HIV-1 Indian clade C envelopes that were tested in this study.

**Table 1:** Neutralization breadth and potency of four bnAbs with distinct gp120 epitope specificities against Indian HIV-1 subtype C

Sl. No	Viruses	IC_50_ (µg/ml)			
		VRC01	CAP256-VRC26.25	PGDM1400	PGT121
1	VB51.J22	0.08	** > 5**	** > 5**	< 0.002
2	VB52.J9	0.77	< 0.002	< 0.002	0.26
3	VB52.J29	1.3	< 0.01	** > 5**	0.09
4	VB52.J30	0.39	< 0.002	< 0.002	0.09
5	VB95.J22	1.3	< 0.01	** > 5**	0.09
6	VB96.J21	0.33	** > 5**	** > 5**	0.04
7	VB96.J44	0.9	3.19	** > 5**	0.04
8	VB97.J10	0.52	** > 5**	3.05	0.09
9	VB97.J15	0.22	** > 5**	0.22	0.12
10	VB98.J1	0.88	< 0.002	< 0.002	0.17
11	VB105.J10	0.2	< 0.002	** > 5**	1.02
12	VB106.J38	0.06	** > 5**	0.32	0.05
13	2.J9	0.37	< 0.002	0.01	** > 5**
14	3-J16	0.06	** > 5**	1.59	< 0.002
15	2–3.J7	0.08	< 0.002	0.01	** > 5**
16	2–3.J4	0.1	< 0.002	0.01	** > 5**
17	2–3.J17	0.09	< 0.002	0.01	** > 5**
18	2–5.J3	0.17	< 0.002	0.03	** > 5**
19	2–5.J11	0.08	< 0.002	0.39	** > 5**
20	3–3.J9	0.17	** > 5**	** > 5**	0.31
21	3–5.J25	0.11	** > 5**	** > 5**	0.01
22	3–5.J38	0.2	0.53	1.01	< 0.002
23	4.J2	0.05	< 0.002	< 0.002	2.14
24	4.J22	0.07	< 0.002	< 0.002	1.43
25	4–2.J45	0.15	< 0.002	0.22	0.03
26	4–2.J46b	0.06	< 0.0046	< 0.002	0.23
27	4–2.J45b	0.05	< 0.002	< 0.002	0.01
28	4–2.J42b	0.06	< 0.002	< 0.002	0.23
29	4–2.J41	0.04	** > 5**	** > 5**	< 0.002
30	4–2.J47b	0.07	0.89	0.07	2.87
31	5.J41	** > 5**	0.05	** > 5**	0.13
32	7.J16	< 0.002	< 0.002	0.01	0.06
33	7.J20	0.03	< 0.002	< 0.002	0.01
34	LT1 07.J1	3.82	< 0.002	0.06	0.07
35	LT1 07.J4	2.93	< 0.002	0.08	< 0.002
36	LT1 07.J26	2.18	< 0.002	< 0.002	< 0.002
37	2–7.J1	0.6	< 0.002	** > 5**	** > 5**
38	11.J25	0.56	0.01	** > 5**	** > 5**
39	11.J28	0.89	3	** > 5**	** > 5**
40	11–3.J9	0.37	< 0.002	< 0.002	< 0.002
41	2–9.J20	0.56	< 0.002	0.1	0.01
42	5.4.J18	** > 5**	< 0.002	0.87	0.07
43	LT1 09.J3	3.05	< 0.002	< 0.002	< 0.002
44	LT1 09.J8	** > 5**	< 0.002	< 0.002	0.03
45	11–5.J12	0.06	< 0.002	< 0.002	< 0.002
46	2–11.J16	0.05	< 0.002	0.01	0.01
47	4–5.J5	0.07	< 0.002	0.04	2.16
48	NISA-N20-J10	0.01	< 0.002	< 0.002	0.01
49	NISA-N20.J14	0.8	< 0.002	< 0.002	0.04
50	NISA-N101.J12	** > 5**	< 0.002	0.03	0.08
51	NISA-N110.J16	0.26	0.4	** > 5**	0.41
52	INDO SA NLR 29.J80	0.37	** > 5**	** > 5**	** > 5**
53	INDO SA NLR 29.J11	0.02	** > 5**	0.13	** > 5**
54	PG37009v2.eJ9	** > 5**	< 0.002	** > 5**	0.22
55	PG37009v2.eJ38	0.06	< 0.002	< 0.002	1.82
56	PG37009v2.eJ58	0.06	< 0.002	0.01	1.61
57	PG37112v2.J5	** > 5**	0.02	** > 5**	0.02
58	PG37112v2.J9	** > 5**	0.02	** > 5**	** > 5**
59	PG37072.J12	0.48	** > 5**	** > 5**	** > 5**
60	PG37072.J16	1.04	0.02	0.07	** > 5**
61	PG37066.J1	** > 5**	0.01	0.04	** > 5**
62	PG37081.J36	** > 5**	< 0.002	< 0.002	0.11
63	PG37087.J39	0.06	** > 5**	** > 5**	** > 5**
64	PG37087.J44	0.05	** > 5**	** > 5**	** > 5**
65	PG37089.J17	0.62	0.02	0.12	** > 5**
66	PG37089.J20	0.27	< 0.01	< 0.01	0.53
67	PG37089.J83	0.26	< 0.01	< 0.01	0.97
68	PG37091.J41	0.06	** > 5**	**> 5**	1.79
69	PG37080.J6A	0.06	** > 5**	** > 5**	** > 5**
70	PG37080v1.J17	0.14	** > 5**	** > 5**	** > 5**
71	PG37080.J158	0.25	2.46	0.09	** > 5**
	% resistant viruses	12.68	23.94	35.21	29.58
	Median IC_50_	0.24	0.0033	0.225	0.095

Virus neutralization assays were done using Env-pseudotyped viruses in TZM-bl cells. IC_50_ values indicate concentrations of IgG that conferred 50% virus neutralization in TZM-bl cells. A starting concentration of 5 µg/ml IgG with threefold dilutions used prior to mixing with the pseudoviruses. Viruses that showed IC50 values > 5 µg/ml are considered resistant and are bold

### Comparing neutralization diversity of Indian clade C with other globally circulating subtypes and recombinants

**Fig. 1:**
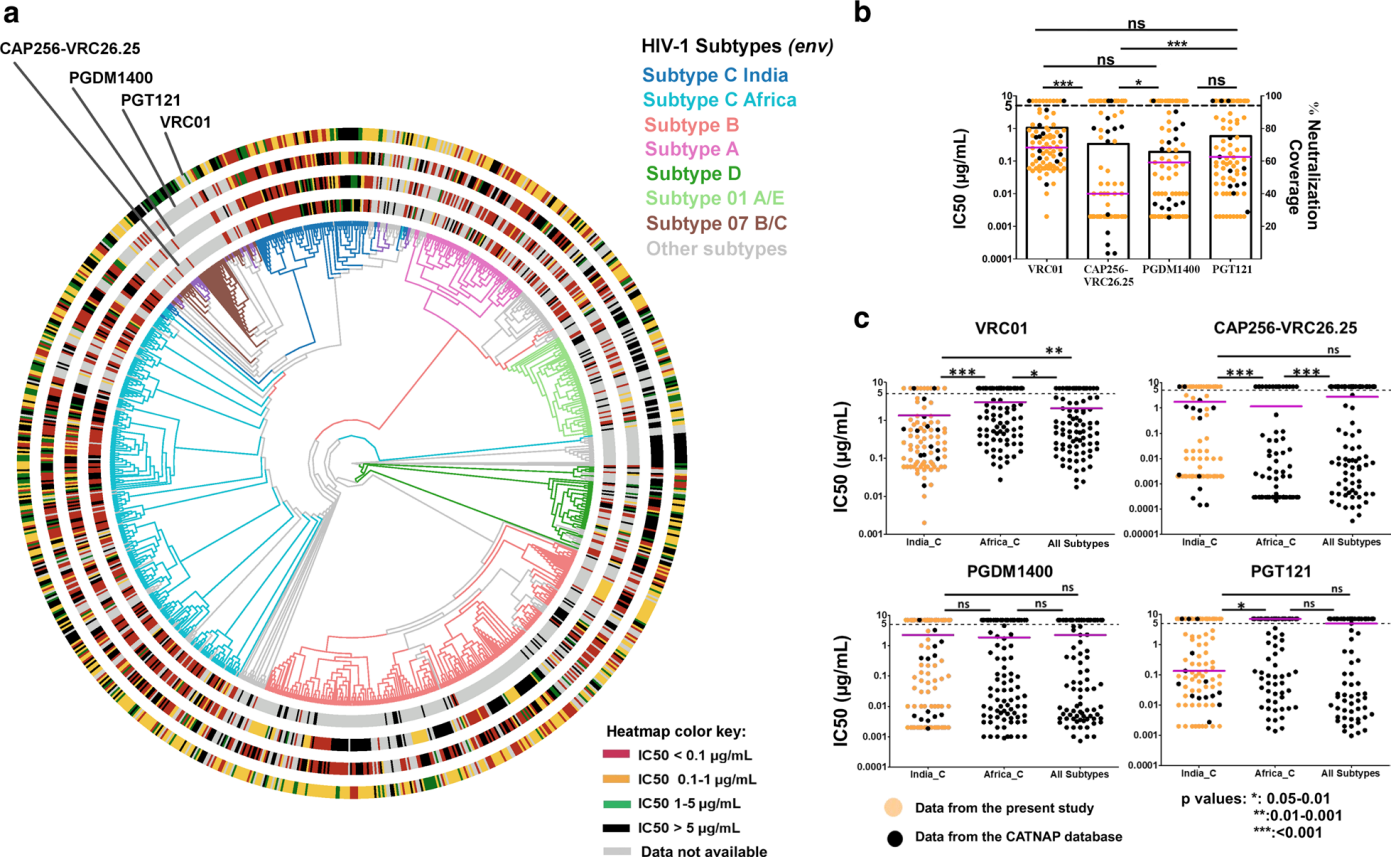
Association of phylogenetic relatedness of env and their responses when expressed as pseudoviruses to the four bnAbs: **a** A phylogenetic tree was constructed for gp160 amino acid sequences of viral clones reported in the present study along with those reported in the CATNAP database (Total N = 1020). The terminal branches of the tree were color-coded based on the subtype as depicted in the color legend. Four heatmaps based on their responses to bnAbs PGDM1400, PGT121, VRC01 and CAP256.-VRC26.25 were overlayed on the phylogenetic tree in the form of concentric tracks one for each bnAb to assess phylogenetic clustering of their IC50 values. IC50 value of 5 µg/mL were considered as neutralization sensitivity threshold. **b** Neutralization potency (scatter plot against left Y axis) was plotted for pseudoviruses expressing 98 subtype C envs (71 from the present study) from India against the four bnAbs. Pink lines indicate median IC50 values. Orange dots represent data generated in the present study while the black dots indicate data retrieved from the CATNAP database. Potency against all the pairs of bnAbs were compared using Mann–Whitney test. p values were illustrated as follows: > 0.05-non-significant, 0.05–0.01- *, 0.01–0.001-** and < 0.001-***. Percent neutralization coverage of all the bnAbs were plotted as a bar chart against the right Y axis. **c** Year matched randomly selected equal number viral clone datasets were retrieved from CATNAP database and their IC50 values to 4 bnAbs (PGT121, PGDM1400, VRC01 and CAP256-VRC26.25 were compared between Subtype C from India, Subtype C pan Africa and Other subtypes (All except C). IC50 value of 5 µg/mL was considered as a threshold of neutralization sensitivity. Statistical comparisons were made between each pair for every antibody using Mann–Whitney test

We next examined whether inter and intra clade *env* diversity have any association with altered neutralization phenotype. For this first we retrieved full length *gp160* sequences of Env-pseudotyped viruses reported in the CATNAP database (www.hiv.lanl.gov) and compared diversity by building a phylogenetic tree (Fig. 1a). As expected, our data very clearly demarcated the distinctness of Indian clade C with non-Indian clade C and other subtypes (Fig. 1a; top left). This observation also corroborates with our earlier finding [39]. The neutralization scores (IC_50_ values) against VRC01, CAP256-VRC26.25, PGDM1400 and PGT121 of most of the same retrieved *gp160* sequences of all subtypes were next obtained from the CATNAP database for the purpose of comparing with that obtained in the current study. A total of 1020 *gp160* sequences were used for the analysis with the following distribution: Subtype C (Pan Africa):290, Subtype C (India): 98 that includes 71 used in the present study, Subtype A: 76, Subtype B: 255, Subtype D: 42, CRF01_AE: 70, CRF07_B/C recombinants: 49, other subtypes and recombinant forms: 140 were assessed for their phylogenetic relatedness using IQtree (HIVdb model, non-parametric 1000 fast bootstrap with aSH-LRT test). Four heatmaps based on their responses to VRC01, CAP256-VRC26.25, PGDM1400 and PGT121 were built based on the phylogenetic tree to reflect the clustering based on their IC_50_ values obtained against each of these Env-pseudotyped viruses. As shown in Fig. 1a, We observed that while VRC01 appeared to be most broad, it was found to be least potent amongst all. CAP256-VRC26.25 appeared to be most potent, however our data also indicates that it has been mostly assessed against HIV-1 subtype C (including one used in the present study). These qualitative observations also reflected upon statistical assessment of viruses from India as indicated in Fig. 1b.

Finally, we compared the overall neutralization sensitivity against these four bnAbs of year matched (1999–2011), randomly selected an equal number of viral *gp160* datasets (n = 85) from CATNAP database with the ones assessed in this current study. The IC_50_ value of 5 µg/ml was considered as a threshold of neutralization sensitivity. As shown in Fig. 1c, the neutralization susceptibility of Indian clade C envelopes to VRC01 were found to be distinct from that observed with the African clade C as well as other subtypes. Interestingly, while the degree of CAP256-VRC26.25 susceptibility between Indian clade C and other subtypes were observed to be comparable, this was found to be significantly different from African clade C. Overall, our data highlights disparate sensitivity of Indian clade C compared to other geographically and phylogenetically distinct HIV-1 subtypes against these four bnAbs.

### Trend analysis of variation in neutralization sensitivity over time at population and individual levels

**Fig. 2:**
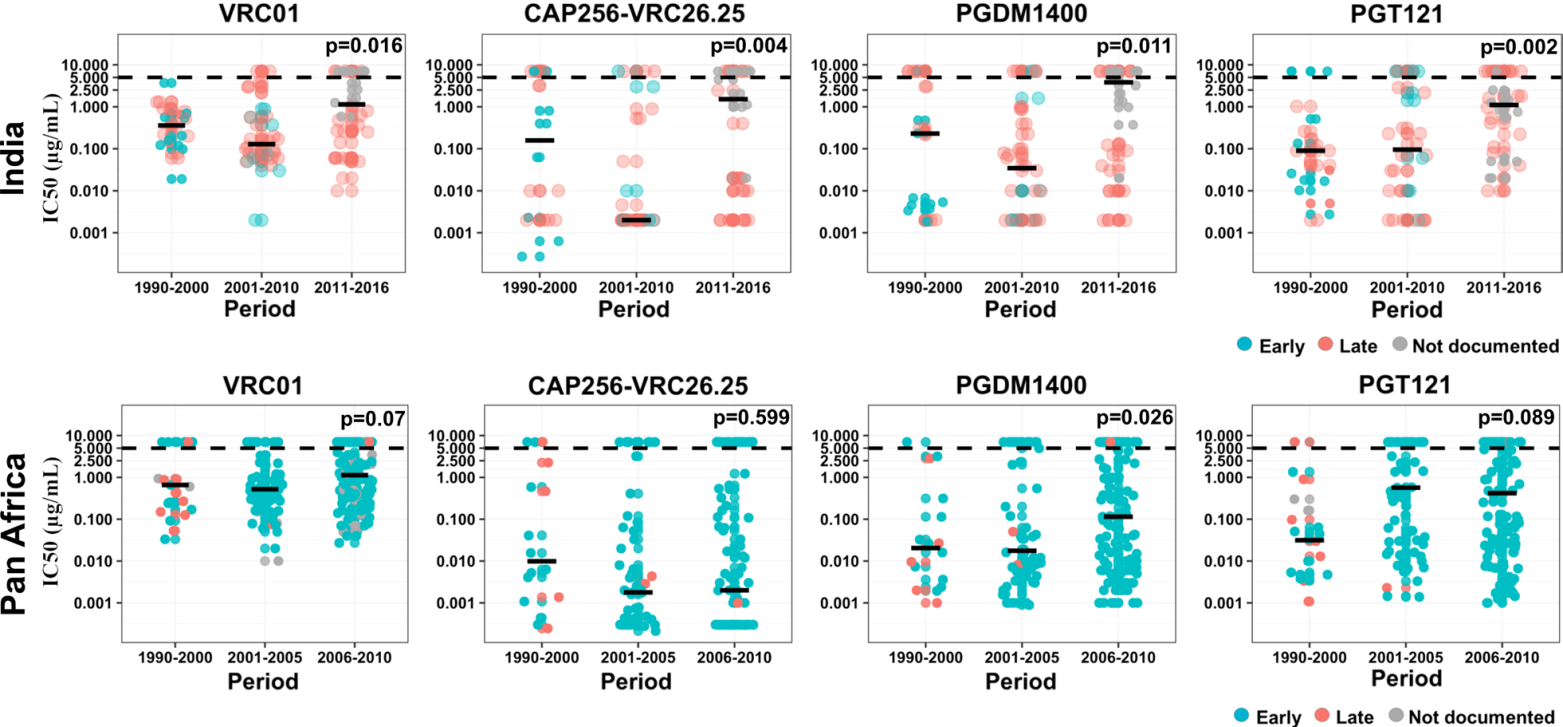
Comparison of trend in bnAb sensitivity across time against VRC01, CAP256-VRC26.25, PGDM1400 and PGT121. **Top panel**. Scatter plots of IC50 values for clones reported in the CATNAP database were grouped as 1990–2000, 2001–2010 and 2011–2016 for viruses from India along with those reported in the present study (indicated by larger dots). **Bottom panel**. Scatter plots of IC50 values for Env-pseudotyped viruses reported in the CATNAP database were grouped as 1990–2000, 2001–2005 and 2006–2010 for envs from Pan-Africa. Data points were color-coded based on the disease stage at sampling of the respective viruses. IC50 value of 5 µg/mL was considered as a threshold of neutralization sensitivity. Statistical assessment of increase in the IC_50_ values was performed with Jonckheere-Terpstra test (JTT)

We next examined the variation in neutralization sensitivity of HIV-1 *env* sequences sampled overtime to these four bnAbs. For this, we grouped IC_50_ values (in three clusters) based on corresponding year of sampling (1990–2000, 2001–2010 and 2011–2016) of the viral *env* sequences. As shown in Fig. 2, the PGDM1400 sensitivity of Env-pseudotyped viruses obtained from both Indian and African population was observed to be decreasing over time. For PGT121 and VRC01 bnAbs, a similar trend was observed in the Indian population. When compared, a gradual increase in resistant neutralization phenotype of African subtypes to PGT121 and VRC01 was also observed over time, it was, however, not found to be significant. Interestingly, an increasing trend in neutralization resistance of Indian clade C envelopes to CAP256-VRC26.25 was observed in contrast to that with envelopes obtained from the African population. Overall, we observed that although a number of Indian clade C envelopes tested in this study were not large enough for an absolute conclusion, our data clearly indicated differences in neutralization susceptibility trend against these four bnAbs overtime between Indian clade C and other subtypes.

**Fig. 3:**
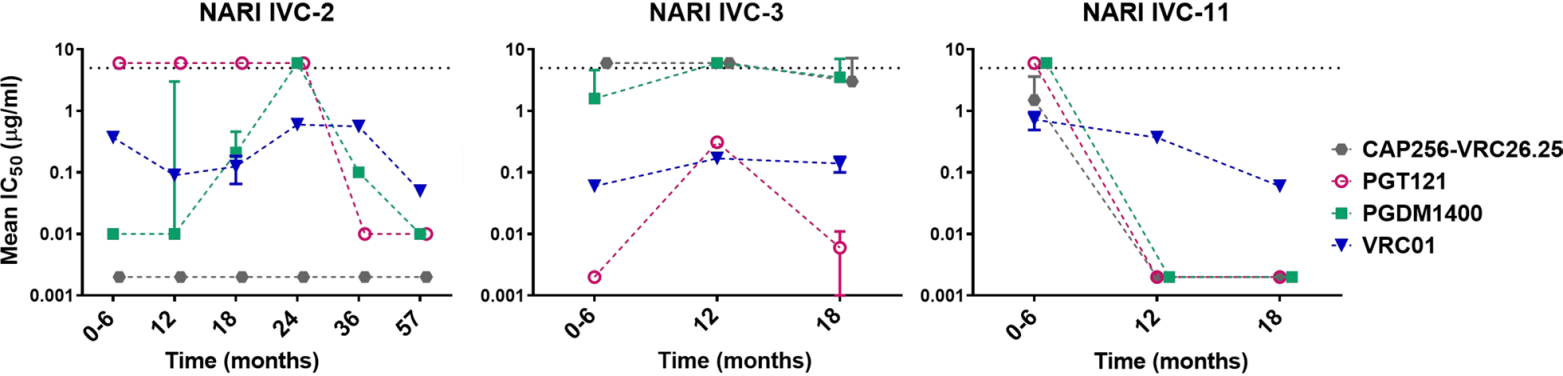
Progression of bnAb responses of pseudoviruses expressing *envs* obtained from longitudinally followed up individuals. Mean IC_50_ values against bnAbs VRC01, CAP256-VRC26.25, PGDM1400 and PGT121 were plotted for viral clones prepared from longitudinally collected samples from three HIV-1 subtype C infected individuals (NARI IVC-2, NARI IVC-3 and NARI IVC-11). IC_50_ value of 5 µg/ml were considered as a threshold of neutralization sensitivity

We next examined the trend of neutralization susceptibility of pseudoviruses expressing primary HIV-1 clade C *envs* obtained from three individuals at different time points to VRC01, CAP256-VRC26.25, PGDM1400 and PGT121. Several of these *envs* were reported earlier by our group [35]. The primary *envs (*complete *gp160* sequences*)* from the donor NARI IVC-2 were obtained at following time point: between 0–6, 12, 18, 24, 36 and 57 months; from donors NARI IVC-3 and NARI IVC-4 at 0–6, 12 and 18 months respectively. As demonstrated in Fig. 3, the trend of susceptibility of pseudoviruses expressing *envs* isolated from these three donors overtime to VRC01, CAP256-VRC26.25, PGDM1400 and PGT121 varied. Out of the three donors, in one (NARI IVC-11), a clear trend of increasing susceptibility of Env-pseudotyped viruses to all the four bnAbs were observed. When tested against PGT121, for NARI IVC-2, pseudoviruses expressing *envs* obtained from baseline to two years, were found to be resistant, while pseudoviruses expressing *envs* obtained from subsequent time points till 57 months were found to be highly sensitive with IC_50_ over 0.01 µg/ml. For all the resistant *envs,* N332 glycan residue was found to be absent. Interestingly, we observed that the *env* (2–11. J16) obtained from 57-month time point was sensitive to PGT121 despite lacking the N332 glycan. While this could possibly be compensated by the presence of a glycan at the 334 position (Additional file 3: Figure S1) as was reported for PGT128 with similar specificity [40], four pseudoviruses expressing *envs* isolated from NARI IVC-2 were found to be resistant to PGT121 despite containing N334. While pseudoviruses expressing *envs* obtained from all time points from this donor were found to be highly sensitive to CAP256-VRC26.25 with IC_50_ < 0.001 µg/ml, no increasing or decreasing trend in susceptibility to VRC01 and PGDM1400 of all the envelopes were observed. For the third donor, NARI IVC-3, envelopes obtained from all the time points were found to be resistant to V1V2 -specific bnAbs CAP256-VRC26.25 and PGDM1400, while they were found to be sensitive to VRC01 and PGT121, however no clear trend in neutralization sensitivity was observed over time. While resistance to different bnAbs was found to be associated with an absence of known target motifs, we found evidence of neutralization resistance in presence of known epitopes that are targets of some bnAbs (Additional file 2: Table S2). Overall, we found that while increasing neutralization resistance trend was observed at the population level, when cross-sectional virus isolates across years were analyzed such a trend was not evident with longitudinal viral *envs* obtained from three followed up Indian donors.

### Predicting optimal bnAb combination using experimental data

**Fig. 4:**
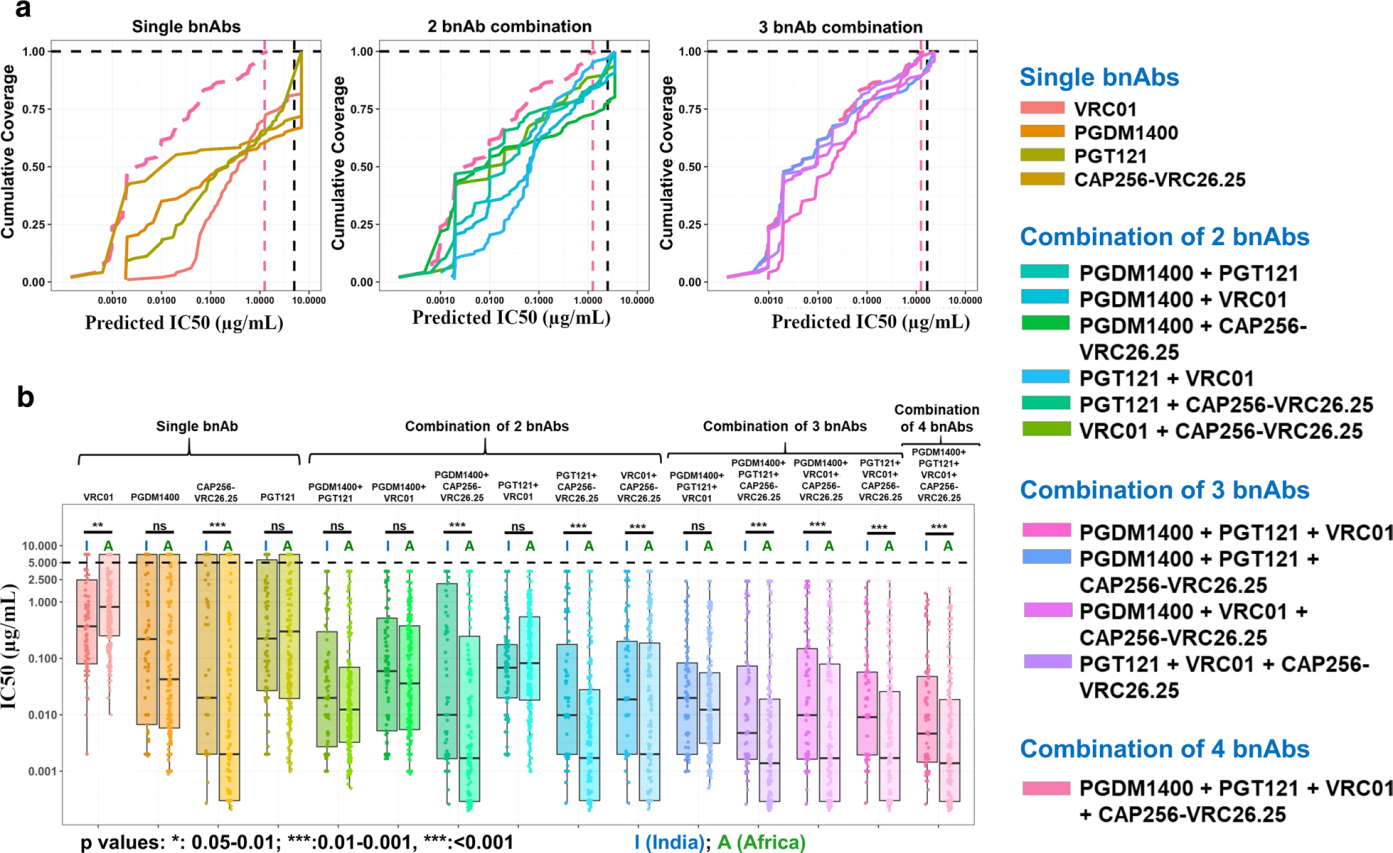
Predictive analysis of effect of bnAb combinations on neutralization breadth and potency of HIV-1 Indian clade C. **a** Cumulative coverage of the virus panel (a fraction between 0 & 1) of 98 clones reported in the present study and CATNAP database from India at various IC50 values for single mAbs and/or mAb combinations with a target Ab concentration of 5 µg/ml. Dashed curve in each of the plots indicates four bnAb combinations. All other combinations are depicted according to the given color codes. Vertical dashed lines in each plot indicate the expected final Ab concentration of four bnAb combination (pink line) and combination under assessment (black line). **b** Predicted geometric IC_50_ values against each bnAb/combination based on experimental IC50 values for viruses from India (N-98, left boxplot in each group) vs Viruses from Africa retrieved from CATNAP (N = 250, right boxplot in each group). Black lines in each plot indicate Median IC50 values. Statistical comparison within each group was performed with Mann–Whitney test

Finally, based on the neutralization data of all the Indian clade C envelopes assessed in this study and neutralization scores of additional Indian clade C *envs* retrieved from CATNAP database (http://www.hiv.lanl.gov), we attempted to predict the optimal combination amongst these four bnAbs that would likely confer maximal neutralization coverage with the highest potency. Towards achieving this, we employed an additive model implemented in the CombiNAber tool (https://www.hiv.lanl.gov/content/sequence/COMBINABER/combinaber.html) to all possible combinations of these four bnAbs (VRC01, CAP256-VRC26.25, PGDM1400 and PGT121). A total of 98 Indian clade C envelopes (71 from the current study and existing neutralization data of 27 additional Indian clade C retrieved from HIV database) for preparing for coverage analysis. As shown in Fig. 4a, the cumulative coverage analysis indicated that each of VRC01, CAP256-VRC26.25, PGDM1400 and PGT121 bnAbs individually could provide 80%, 70%, 65% and 74%coverage respectively at IgG concentration of equal to or below 5 µg/ml. When assessed for the minimum combination that would provide maximal neutralization coverage, two combinations of three bnAbs each (VRC01 + CAP56-VRC26.25 + PGT121 and PGDM1400 + CAP256-VRC26.25 + PGT121) appears to provide 100% cumulative coverage at total IgG concentration of 5 µg/ml or less as predicted by CombiNAber. In parallel, we also examined whether these two bnAb combinations of three bnAbs could achieve the maximal neutralization coverage at low doses. Towards this, the median IC_50_ values of Indian clade C envelopes (n = 98) obtained against single and different combinations of all the four bnAbs were obtained and the coverage versus potency assessment was statistically validated by Mann–Whitney test on the dataset obtained by implementation of the additive model by the CombiNAber tool with a target limit of IgG concentration of 5 µg/ml. As shown in Fig. 4b, we observed that a combination of CAP256-VRC26.25 + PGDM1400 and PGT121 could achieve near 100% breadth with the highest potency (Fig. 4b). This was found to be strikingly different from what we observed with African subtype C, where two combinations of each of these four bnAbs appeared to provide near 100% neutralization coverage with maximal potency. For all the different combination we assessed, we compared the median IC_50_ obtained in our study with that reported for African clade C envelopes (n = 250) by retrieving neutralization IC_50_ values from CATNAP database (http://www.hiv.lanl.gov). As expected, we found that the same combinations of three bnAbs (VRC01 + CAP256-VRC26.25 + PGT121 and PGDM1400 + CAP256-VRC26.25 + PGT121) demonstrated maximal coverage with geometric cumulative mean IC_50_ of 0.0109 and 0.01137 µg/ml respectively and are observed to be comparable to what was observed with the combination of all four bnAbs. Of interest, when compared with African clade C envelopes, we observed that a combination of two bnAbs that commonly include CAP256-VRC26.25 (such as CAP256-VRC26.25 + PGDM1400, CAP256-VRC26.25 + VRC01 and CAP256-VRC26.25 + PGT121) appear to provide comparable coverage with significantly lower doses than what we observed with the three bnAb combinations with Indian clade C viruses (Fig. 4b). This is likely because CAP256-VRC26.25 was found to demonstrate better neutralization coverage of African clade C with increased potency compared to what we observed with Indian clade C viruses.

## Discussion

Understudied and unexplored population and clade-specific HIV-1 sequence diversity presents a formidable challenge that is likely to play a major hindrance in designing of effective antibody-mediated intervention strategies. Subtype C being the most predominant subtype circulating majorly in Africa and India, a greater understanding of the antigenic and neutralization diversity of HIV-1 subtype C *env* would facilitate understanding of optimal bnAb combination that could potentially overcome region-specific the intra-clade C diversity. Our recent work has demonstrated genotypic distinctness of HIV-1 subtype C envelope (gp120) sequences reported from Indian and African epidemics [39]. In that study, we observed these sequences to be distinct in terms of variable loop lengths (V1, V2 and V4), an abundance of potential N-linked glycosylation sites and entropy at bnAb contact sites (including PGDM1400 and CAP256-VRC26.25). Through prediction algorithms, we explored the impact of these diverging signatures on sensitivity to bnAbs and predicted appreciable differences. Through this present study, we attempted to experimentally elucidate the distinct neutralization phenotype of primary HIV-1 subtype C obtained from Indian patients to four key bnAbs that not only have demonstrated considerable breadth, but also amongst those which are farthest into clinical development and testing. When analyzed against pseudoviruses expressing 71 primary *envs*, we observed that while VRC01 demonstrated maximum breadth, PGDM1400 showed the least amongst the four. Conversely, CAP256-VRC26.25 was found to neutralize over 76% viruses with maximal potency amongst others. Interestingly, while the neutralization resistance of the viruses to PGDM1400 was majorly found to be associated with known substitutions as found in the HIV database (http://www.hiv.lanl.gov), for some resistant envelopes, no known resistant motifs were found, indicating plausible underlying unknown mechanism associated with PGDM1400 resistance. Furthermore, while CAP256-VRC26.25 and PGDM1400 target epitopes on the V1V2 loop, Indian clade C envelopes were found to be more susceptible to CAP256-VRC26.25 with fewer resistant signatures compared to PGDM1400. This is an interesting and perhaps an important observation which warrants the need to screen for additional significantly large population-based study to understand the frequency of occurrence of signature motifs and/or substitutions associated with neutralization resistance. This is particularly important while choosing bnAbs targeting V1V2 region for clinical use as V1V2 region represents the most hypervariable region and subtle changes in loop length, glycosylation patterns and substitutions will compromise with the antibody-mediated virus neutralization efficacy [41]. Indeed, a significant difference in neutralization breadth and potency was observed when we compared the sensitivity of Indian subtype C with that of African subtype C based on the existing data. Interestingly though, while comparable sensitivity to PGDM1400 was observed between Indian and African subtype C viruses, significant differences were observed when sensitivities to VRC01, CAP256-VRC26.25 and PGT121 were assessed. This observation further highlights the variation in population and geography based *env* genetic diversity associated with neutralization diversities.

Trend analysis at the population level with limited data sets indicated a sense of increasing resistance to all the four bnAbs tested in this study with envelopes isolated from Indian patients between the years 2011–2016. This could possibly be linked to the differences in host characteristics and varied genetic bottlenecks which contribute to altered virus and antibody evolution pathways in the course of natural infection. Our observation while found reasonably comparable to what was observed with African subtype C, except for CAP256-VRC26.25, we noted that most if not all the African C envelopes were obtained from acute/early infection. In this line, given this was an observational study, we also hypothesize that the stage of the disease, origin of the *env*, and the possible geographic differences (within and outside India) could potentially influence on the neutralization phenotypes to draw any robust conclusion. At the individual level, while an increasing susceptibility trend was observed with PGT121, no consistent trend was observed with other three bnAbs. While we tested *envs* obtained from follow up visits of only three individuals, it would be important to study *envs* obtained from more number of individuals in the same follow-up setting. Such exercise would possibly help understand optimal combination of bnAbs that would be suitable for prophylaxis or early treatment versus the combination that would be more suitable to treat at the late disease stage.

One of the approaches that has widely been considered to provide maximal neutralization breadth at low dose and overcoming virus escape (particularly in the context of antibody-mediated treatment) is a combination of bnAbs having distinct epitope specificities. Based on our experimental data, we observed that combination of two V1V2 directed (CAP256-VRC26.25, PGDM1400) and one V3 supersite-directed (PGT121) bnAbs could achieve near 100% neutralization coverage with maximal potency. Intriguingly, this observation was found to be distinct to what we observed with African subtype C where combinations of two bnAbs could possibly provide near 100% neutralization coverage at the highest potency. Our observation further highlighted neutralization diversity between Indian and African subtype C. It is to be noted that while the majority of the African clade C *env* sequences analyzed in this study were derived from acute and early infection cases (http://www.hiv.lan.gov), majority of the Indian subtype C *env* sequences used in the present study were obtained from individuals post 6 months of infection and at late disease stages. Assessment of late disease stages from both regions would be important to design bnAb based therapeutic strategies. While not many Indian subtype C *env* sequences from acute/early infection are available, we believe that it will be very important to obtain such sequences that will be necessary to define, select and optimize bnAbs and their combination for developing antibody-mediated prevention strategies. Several factors might account for this variability. As a result of the continuous drift of the HIV-1 genome and epitopes, it is possible that divergent subtypes follow separate evolution pathways that may impact their degree of susceptibility to different bnAbs. This may also be due to the population-specific diverse and distinct selection pressure. Our hypothesis is supported by the fact that different classes of bnAbs isolated from HIV-1 infected individuals were influenced by both the infecting subtypes [23] and also by the ethnic origin [24, 42]. In addition, the possibility of increasing resistance to bnAbs as observed [25, 26] in recent studies over the course of the epidemic warrants the necessity for continued surveillance of virus evolution that would impact the ability of the promising bnAbs mediated neutralization coverage of region-specific HIV-1 diversities.

## Conclusion

In conclusion, our data indicated disparate neutralization diversities of HIV-1 subtype C when assessed against four bnAbs with distinct epitope specificities and those that are amongst the furthest into the clinical development and testing. This observation necessitates the urgent need to compare a large number of *env* sequences across different geographies, risk groups and key populations to understand how disparate intra-clade genetic diversity could potentially impact virus neutralization. This will inform the choice of the bnAb combinations that would be suitable for optimal coverage of the region-specific HIV-1 circulating subtypes for prevention and treatment over and above the existing antiretroviral drugs.

## Methods

### Ethics statement

The plasma and PBMC samples used for amplification of complete *env* genes by PCR were collected following respective institutional ethical approvals.

### Plasmids, antibodies and cell lines

Plasmid DNA encoding HIV-1 *gp160* sequences published earlier are listed in Table 1. Plasmid DNA encoding heavy and light variable IgG chain sequences of the following monoclonal antibodies: PGT121, PGDM1400 and VRC01 were obtained from the IAVI neutralizing antibody center at the Scripps Research and CAP256.VRC26.25 was kindly provided by Prof Lynn Morris, National Institute of Communicable Diseases (NICD), Johannesburg, South Africa under an ongoing collaborative project. TZM-bl cells were obtained from the NIH AIDS Research Program. HEK-293 T cell line was obtained from American Type Culture Collection (ATCC).

### Amplification and cloning of gp160

Full length *env* (*gp160*) genes were PCR amplified from HIV + plasma samples with slight modification as described previously [37]. Briefly, viral RNA was extracted using a high-pure viral RNA kit (Roche Inc.) by following manufacturer’s protocol, and cDNA was prepared by reverse transcription-PCR (RT-PCR) using a Superscript III first-strand synthesis kit (Invitrogen Inc.). The primer used for cDNA synthesis was EnvR1 5′-GCACTCAAGGCAAGCTTTATTGAGGCTT -3′ (HXB2: 9605–9632). Rev-Env gp160 cassette were amplified from the cDNA product using La Taq high fidelity DNA polymerase in the 1st round (Takara Bio Inc.) and PrimeSTAR GXL high fidelity DNA polymerase (Takara Bio Inc.) in the second round. The primers used for the 1^st^ round were EnvF1: 5′- AGARGAYAGATGGAACAAGCCCCAG-3′ (HXB2: 5550–5574) and EnvRP2: 5′-GTGTGTAGTTCTGCCAATCAGGGAA -3′ (HXB2: 9157–9181) while for the second round were Env IF: 5′-CACCGGCTTAGGCATCTCCTATGGCAGGAAGAA -3′ (HXB2: 5950–5982) and Env IR: 5′-TATCGGTACCAGTCTTGAGACGCTGCTCCTACTC -3′ (HXB2: 8882–8915). PCR condition followed in the first round was initial denaturation of 94 °C for 2 min followed by 12 cycles of 94 °C for10 secs, 60 °C for 30 secs, 68 °C for 3 min, 23 cycles of 94 °C for 10 secs, 55 °C for 30 s, 68 °C for 3 min with final extension of 68 °C for 10 min. PCR condition followed in the second round was initial denaturation of 94 °C for 2 min followed by 12 cycles of 94 °C for 10 secs, 62 °C for 30 s, 68 °C for 3 min, 23 cycles of 94 °C for 10 secs, 60 °C for 30secs, 68 °C for 3 min with a final extension of 68 °C for 10 min. The *gp160* amplicons were purified and ligated into pcDNA 3.1/V5-His-TOPO (Invitrogen Inc.) vector or pSVIII as described before [35].

### Sequence analysis

The gp160 amino acid sequences generated in the present study, those published in the GenBank earlier as well as those derived from the CATNAP database (supplementary information) were aligned to each other using Muscle v3.8.1551. The resulting alignment was curated using BioEdit sequence alignment editor v7.2.5 [43]. Variable loop regions for V1 (131–157: HXB2 numbering), V2 (158–196), V3 (296–331), V4 (386–417) and V5 (460–469) were retrieved from amino acid alignments with Extractalign implementation of the Emboss package [44]. Each of the loop datasets were then processed with custom bash/awk scripts to generate length statistics. Potential N-linked glycosylation sites were predicted using N-Glycosite tool available at the LANL HIV database [45]. Statistics regarding the antibody resistance-associated signature residues were generated as described recently [39]. A phylogenetic tree was generated for all the HIV-1 viral clone sequences available in the CATNAP database along with those generated in the present study (Total N = 1020) with iqtree under ‘HIVb’ model with estimated Ƴ parameters and number of invariable sites [46]. The robustness of the tree topology was further assessed by SH-aLRT as well as 1000 ultrafast bootstrap replicates implemented in iqtree. The tree as well as neutralization IC50 (µg/ML) values for each of the sequences against bnAbs PGDM1400, PGT121, VRC01 and CAP256-VRC26.25 were plotted with ‘ggtree’ package in R [47, 48]. IC50 values for year matched randomly selected equal number (N = 85) were compared for HIV-1 clade C viral clones reported from India (including this study), Africa as well as other subtypes using Mann–Whitney test. Trend analysis for change in viral sensitivity to bnAbs VRC01, PGDM1400, CAP256-VRC26.25 and PGT121 was further performed. To achieve this, IC50 values for all Indian viral clones were grouped into three-time periods as per their year of sampling (1990–2000, 2001–2010 and 2011–2016) and compared with Jonckheere Terpstra test (JTT). CombiNAber (http://www.hiv.lanl.gov) [25], was used to predict optimal antibody combination for maximum neutralization of viral clones from India (N = 98) and from Africa (N-250) using their experimentally determined IC50 values. Target IgG concentration of 5 µg/mL was considered as a threshold followed by implementation of the additive model in the CombiNAber tool to obtain geometric mean IC_50_ values for each combination. Sensitivity to each of these bnAbs combinations was further compared between Indian and African viruses using Mann–Whitney test. statistical computing software (v3.4.0) and R studio v1.0.143 [49, 50]. Statistical analysis for neutralization breadth and potency were done using GraphPad Prism version 5.01 for Windows, GraphPad Software, San Diego California USA. Scatter plots for bnAb potency distribution across geographically distinct populations as well as those from longitudinally collected samples were assessed statistically and plotted in GraphPad Prism v5.01.

### Preparation of Env-pseudoviruses

Pseudotyped viruses were prepared by the co-transfection of *env*-expressing plasmid DNA along with the plasmid DNA expressing HIV-1 genes with premature stop codon for *env* (pSG3ΔEnv) into 293 T cells in 6-well tissue culture plates using FuGENE6 transfection reagent kit (Promega Inc.). Cell supernatants containing pseudotyped viruses were harvested at 48 h post transfection and subsequently stored at -80° C until use. The virus infectivity was measured using TZM-bl reporter cells by addition of pseudoviruses containing DEAE-dextran (25 µg/ml) in 96-well microtiter plates, and the viral titers were determined by measuring the luciferase activity using Britelite luciferase substrate (PerkinElmer Inc.) with a Victor X2 luminometer (PerkinElmer Inc.).

### Neutralization assay

Neutralization assays were carried out using TZM-bl cells as described before [37]. Briefly, Env-pseudotyped viruses were pre-incubated in 96-well tissue culture plates with various concentrations of bnAbs (IgG) for an hour at 37° C in a CO_2_ incubator under humidified conditions. Subsequently, 1 × 10^4^ TZM-bl cells were added to the mixture in the presence of 25 µg/ml DEAE-dextran (Sigma, Inc.). The plates were further incubated for 48 h. The degree of virus neutralization was assessed by measuring reduction in relative luminescence units (RLU) in a luminometer (Victor X2; PerkinElmer Inc.).

## Supplementary Information

**Additional file 1: Table S1**. History of year of collection of clinical samples, disease stages and *env* genetic properties.

**Additional file 2: Table S2**. Mapping key amino acid substitutions associated with resistance to the bnAbs.

**Additional file 3: Figure S1.** Amino acid sequence alignment of V3 region of the autologous HIV-1 *env* obtained from NARI IVC-2, NARI IVC-3 and NARI IVC-11 collected at different time points in a span of 2006–2011 to denote the frequency of occurrence of N334 glycosylation.

## Data Availability

The dataset(s) supporting the conclusions of this article is (are) included within the article (and its additional file(s). The complete nucleotide sequences of the following *envs*: 4.2.J45b, 2–7.J1, 2–9.J20, 2–11.J16, NISA-N20-J10, NISA-N20.J14, NISA-N101.J12, INDO SA NLR 29.J80, INDO SA NLR 29.J11, PG37009v2.eJ9, PG37009v2.eJ38, PG37009v2.eJ58, PG37112v2.J5, PG37112v2.J9, PG37072.J12, PG37072.J16, PG37066.J1, PG37081.J36, PG37087.J39, PG37087.J44, PG37089.J17, PG37089.J20, PG37089.J83, PG37091.J41, PG37080.J6A, PG37080v1.J17, PG37080.J158 are being submitted to the GenBank.

## Abbreviations

HIV: Human Immunodeficiency Virus
bnAb: Broadly neutralizing monoclonal antibodies
[SH]-aLRT: Shimodaira-Hasegawa approximate likelihood ratio test
CATNAP: Compile, Analyze and Tally NAb Panels
ARV: Antiretroviral drugs
