# Supplementary material for: Neutralization diversity of HIV-1 Indian subtype C envelopes obtained from cross sectional and followed up individuals against broadly neutralizing monoclonal antibodies having distinct gp120 specificities
Source: Retrovirology. 2021 May 14;18:12. doi: 10.1186/s12977-021-00556-2 (PMC8120817; doi:10.1186/s12977-021-00556-2)
Supplement: Supplementary file 1 — Additional file 1: Table S1. History of year of collection of clinical samples, disease stages and env genetic properties. [file 12977_2021_556_MOESM1_ESM.docx]

**Table S1.** History of year of collection of clinical samples, disease stages and *env (gp160)* genetic properties.

| **Sl. No.** | **Viruses** | **Year of collection (1999-2014)** | **Disease stage** | **Source** | **GenBank ID** | **PNLG** | **V1 loop** | **V2 loop** | **V1V2 loop** | **V3 loop** | **V4 loop** | **V5 loop** | **References** |
| --- | --- | --- | --- | --- | --- | --- | --- | --- | --- | --- | --- | --- | --- |
| 1 | VB51.J22 | 1999 | Late | PBMC | EU622001 | 30 | 25 | 48 | 73 | 37 | 32 | 12 | Gharu et al. *Virus Research* 158 (2011) 216–224 |
| 2 | VB52.J9 | 1999 | Late | PBMC | EU760888 | 32 | 32 | 42 | 74 | 37 | 28 | 13 | Gharu et al. *AIDS Res Human Retrovirology*, 27(2):211-219, 2011 |
| 3 | VB52.J29 | 1999 | Late | PBMC | EU760889 | 30 | 33 | 42 | 75 | 37 | 28 | 13 | Gharu et al. *AIDS Res Human Retrovirology*, 27(2):211-219, 2011 |
| 4 | VB52.J30 | 1999 | Late | PBMC | EU760890 | 30 | 33 | 41 | 74 | 37 | 28 | 13 | Gharu et al. *AIDS Res Human Retrovirology*, 27(2):211-219, 2011 |
| 5 | VB95.J22 | 1999 | Late | PBMC | EU622004 | 28 | 20 | 40 | 60 | 37 | 27 | 13 | Gharu et al. *Virus Research* 158 (2011) 216–224 |
| 6 | VB96.J21 | 2000 | Late | PBMC | EU521728 | 30 | 19 | 45 | 64 | 37 | 40 | 13 | Gharu et al. *Virus Research* 158 (2011) 216–224 |
| 7 | VB96.J44 | 2000 | Late | PBMC | EU622005 | 31 | 19 | 45 | 64 | 37 | 43 | 13 | Gharu et al. *Virus Research* 158 (2011) 216–224 |
| 8 | VB97.J10 | 2000 | Late | PBMC | EU622007 | 31 | 28 | 50 | 78 | 37 | 32 | 13 | Gharu et al. *Virus Research* 158 (2011) 216–224 |
| 9 | VB97.J15 | 2000 | Late | PBMC | EU622008 | 32 | 28 | 50 | 78 | 37 | 30 | 12 | Gharu et al. *Virus Research* 158 (2011) 216–224 |
| 10 | VB98.J1 | 2000 | Late | PBMC | EU622009 | 28 | 20 | 42 | 62 | 37 | 27 | 13 | Gharu et al. *Virus Research* 158 (2011) 216–224 |
| 11 | VB105.J10 | 2000 | Late | PBMC | EU521729 | 28 | 30 | 39 | 69 | 37 | 36 | 13 | Gharu et al. *Virus Research* 144 (2009) 306–314 |
| 12 | VB106.J38 | 2000 | Late | PBMC | EU622015 | 28 | 20 | 40 | 60 | 37 | 29 | 11 | Gharu et al. *Virus Research* 158 (2011) 216–224 |
| 13 | 2.J9 | 2006 | Early | PBMC | EU908215.1 | 30 | 28 | 46 | 72 | 37 | 28 | 11 | Ringe et al. *Retrovirology* 2010, 7:76 |
| 14 | 3.J16 | 2006 | Early | PBMC | EU908217.1 | 27 | 22 | 41 | 63 | 37 | 33 | 12 | Ringe et al. *Retrovirology* 2010, 7:80 |
| 15 | 2-3. J7 | 2007 | Late | PLASMA | GU945308.1 | 29 | 25 | 45 | 70 | 37 | 28 | 10 | Ringe et al. *Retrovirology* 2010, 7:76 |
| 16 | 2-3. J4 | 2007 | Late | PLASMA | GU945307.1 | 30 | 25 | 45 | 69 | 37 | 28 | 10 | Ringe et al. *Retrovirology* 2010, 7:77 |
| 17 | 2-3. J17 | 2007 | Late | PLASMA | GU945309.1 | 28 | 25 | 40 | 64 | 37 | 28 | 10 | Ringe et al. *Retrovirology* 2010, 7:78 |
| 18 | 2-5. J3 | 2007 | Late | PLASMA | GU945311.1 | 31 | 25 | 46 | 70 | 37 | 28 | 10 | Ringe et al. *Retrovirology* 2010, 7:79 |
| 19 | 2-5. J11 | 2007 | Late | PLASMA | GU945312.1 | 29 | 25 | 45 | 69 | 37 | 28 | 10 | Ringe et al. *Retrovirology* 2010, 7:80 |
| 20 | 3-3. J9 | 2007 | Late | PLASMA | GU945313.1 | 28 | 17 | 41 | 58 | 37 | 33 | 11 | Ringe et al. *Retrovirology* 2010, 7:81 |
| 21 | 3-5. J25 | 2007 | Late | PLASMA | GU945314.1 | 29 | 15 | 41 | 56 | 37 | 33 | 11 | Ringe et al. *Retrovirology* 2010, 7:82 |
| 22 | 3-5. J38 | 2007 | Late | PLASMA | GU945315.1 | 31 | 20 | 41 | 61 | 37 | 33 | 11 | Ringe et al. *Retrovirology* 2010, 7:83 |
| 23 | 4.J2 | 2007 | Early | PBMC | EU908218 | 30 | 22 | 40 | 62 | 37 | 30 | 12 | Ringe et al. *Retrovirology* 2010, 7:84 |
| 24 | 4.J22 | 2007 | Early | PBMC | EU908219.1 | 30 | 22 | 40 | 62 | 37 | 30 | 12 | Ringe et al. *Retrovirology* 2010, 7:85 |
| 25 | 4-2.J45 | 2007 | Late | PLASMA | GU945317.2 | 27 | 19 | 40 | 59 | 37 | 30 | 12 | Ringe et al. *Retrovirology* 2010, 7:86 |
| 26 | 4-2.J46b | 2007 | Late | PLASMA | GU945319.2 | 30 | 24 | 40 | 64 | 37 | 30 | 12 | Ringe et al. *Retrovirology* 2010, 7:87 |
| 27 | 4-2.J45b | 2007 | Late | PLASMA | **This study** | 26 | 19 | 40 | 59 | 37 | 30 | 12 | Ringe et al. *Retrovirology* 2010, 7:88 |
| 28 | 4-2.J42b | 2007 | Late | PLASMA | GU945318.2 | 27 | 24 | 40 | 64 | 37 | 30 | 12 | Ringe et al. *Retrovirology* 2010, 7:89 |
| 29 | 4-2.J41 | 2007 | Late | PLASMA | GU945316.2 | 27 | 19 | 40 | 59 | 37 | 30 | 12 | Ringe et al. *Retrovirology* 2010, 7:90 |
| 30 | 4-2. J47b | 2007 | Late | PLASMA | GU945320.1 | 27 | 19 | 40 | 59 | 37 | 30 | 12 | Ringe et al. *Retrovirology* 2010, 7:91 |
| 31 | 5.J41 | 2007 | Early | PBMC | EU908221.1 | 29 | 21 | 51 | 72 | 37 | 31 | 11 | Ringe et al. *Retrovirology* 2010, 7:93 |
| 32 | 7.J16 | 2007 | Early | PLASMA | EU908222.1 | 28 | 23 | 41 | 64 | 37 | 30 | 11 | Ringe et al. *Retrovirology* 2010, 7:95 |
| 33 | 7.J20 | 2007 | Early | PLASMA | EU908223.1 | 26 | 23 | 41 | 64 | 37 | 30 | 11 | Ringe et al. *Retrovirology* 2010, 7:96 |
| 34 | LT1 07.J1 | 2007 | Late | PLASMA | JN400529 | 27 | 20 | 42 | 62 | 37 | 29 | 11 | Mukhopadhyay, S. *et al.* 2012. *AIDS Res Human Retrovirol,* 28: 739-745 |
| 35 | LT1 07. J4 | 2007 | Late | PLASMA | JN400530 | 27 | 20 | 42 | 62 | 37 | 29 | 11 | Mukhopadhyay, S. et al. 2012. *AIDS Res Human Retrovirol,* 28: 739-745 |
| 36 | LT1 07. J26 | 2007 | Late | PLASMA | JN400531 | 27 | 20 | 42 | 62 | 37 | 29 | 11 | Mukhopadhyay, S. et al. 2012. *AIDS Res Human Retrovirol,* 28: 739-745 |
| 37 | 2-7. J1 | 2008 | Late | PLASMA | **This study** | 30 | 25 | 45 | 66 | 37 | 28 | 12 | **This study** |
| 38 | 11.J25 | 2008 | Early | PBMC | EU908224.1 | 27 | 18 | 46 | 64 | 37 | 27 | 12 | Ringe et al. *Retrovirology* 2010, 7:97 |
| 39 | 11.J28 | 2008 | Early | PBMC | EU908225.1 | 27 | 18 | 46 | 64 | 37 | 27 | 12 | Ringe et al. *Retrovirology* 2010, 7:98 |
| 40 | 11-3. J9 | 2008 | Late | PLASMA | GU945330.1 | 27 | 17 | 46 | 63 | 37 | 27 | 9 | Ringe et al. *Retrovirology* 2010, 7:99 |
| 41 | 2-9. J20 | 2009 | Late | PLASMA | **This study** | 31 | 22 | 43 | 65 | 37 | 28 | 11 | **This study** |
| 42 | 5-4. J18 | 2009 | Late | PLASMA | GU945327.1 | 30 | 21 | 51 | 72 | 37 | 34 | 11 | Ringe et al. *Retrovirology* 2010, 7:94 |
| 43 | LT1_09.J3 | 2009 | Late | PLASMA | JN400534 | 27 | 18 | 45 | 63 | 37 | 22 | 11 | Mukhopadhyay, S. et al. 2012. *AIDS Res Human Retrovirol*, 28: 739-745 |
| 44 | LT1_09. J8 | 2009 | Late | PLASMA | JN400536 | 27 | 18 | 45 | 63 | 37 | 22 | 11 | Mukhopadhyay, S. et al. 2012*. AIDS Res Human Retrovirol*, 28: 739-745 |
| 45 | 11-5. J12 | 2009 | Late | PLASMA | GU945332.1 | 28 | 18 | 46 | 64 | 37 | 27 | 12 | Ringe et al. *Retrovirolog*y 2010, 7:100 |
| 46 | 2-11. J16 | 2011 | Late | PLASMA | **This study** | 28 | 22 | 43 | 65 | 37 | 20 | 11 | **This study** |
| 47 | 4-5. J5 | 2011 | Late | PLASMA | GU945321.1 | 28 | 19 | 40 | 59 | 37 | 26 | 12 | Ringe et al. *Retrovirology* 2010, 7:92 |
| 48 | NISA-N20-J10 | 2011 | Late | PLASMA | **This study** | 30 | 23 | 42 | 63 | 37 | 39 | 13 | **This study** |
| 49 | NISA-N20.J14 | 2011 | Late | PLASMA | **This study** | 30 | 29 | 46 | 73 | 39 | 34 | 12 | **This study** |
| 50 | NISA-N101.J12 | 2011 | Late | PLASMA | **This study** | 24 | 24 | 45 | 67 | 37 | 32 | 14 | **This study** |
| 51 | NISA-N110.J16 | 2011 | Late | PLASMA | **This study** | 27 | 37 | 40 | 75 | 37 | 34 | 17 | **This study** |
| 52 | INDO SA NLR 29. J80 | 2011 | Late | PLASMA | **This study** | 28 | 32 | 43 | 75 | 37 | 33 | 13 | **This study** |
| 53 | INDO SA NLR 29. J11 | 2011 | Late | PLASMA | **This study** | 21 | 30 | 42 | 72 | 37 | 32 | 14 | **This study** |
| 54 | PG37009v2.eJ9 | 2014 | Late | PLASMA | **This study** | 23 | 15 | 38 | 53 | 37 | 24 | 9 | **This study** |
| 55 | PG37009v2.eJ38 | 2014 | Late | PLASMA | **This study** | 25 | 23 | 43 | 66 | 37 | 23 | 10 | **This study** |
| 56 | PG37009v2.eJ58 | 2014 | Late | PLASMA | **This study** | 24 | 23 | 43 | 66 | 37 | 23 | 10 | **This study** |
| 57 | PG37112v2.J5 | 2014 | Late | PLASMA | **This study** | 29 | 36 | 41 | 77 | 37 | 42 | 14 | **This study** |
| 58 | PG37112v2.J9 | 2014 | Late | PLASMA | **This study** | 29 | 36 | 41 | 77 | 37 | 45 | 14 | **This study** |
| 59 | PG37072.J12 | 2014 | Late | PLASMA | **This study** | 29 | 28 | 42 | 70 | 37 | 27 | 10 | **This study** |
| 60 | PG37072.J16 | 2014 | Late | PLASMA | **This study** | 28 | 28 | 42 | 70 | 37 | 27 | 10 | **This study** |
| 61 | PG37066.J1 | 2014 | Late | PLASMA | **This study** | 27 | 40 | 40 | 80 | 37 | 33 | 10 | **This study** |
| 62 | PG37081.J36 | 2014 | Late | PLASMA | **This study** | 27 | 29 | 46 | 73 | 37 | 30 | 11 | **This study** |
| 63 | PG37087.J39 | 2014 | Late | PLASMA | **This study** | 33 | 36 | 41 | 77 | 37 | 35 | 12 | **This study** |
| 64 | PG37087.J44 | 2014 | Late | PLASMA | **This study** | 33 | 39 | 42 | 79 | 37 | 35 | 13 | **This study** |
| 65 | PG37089.J17 | 2014 | Late | PLASMA | **This study** | 29 | 40 | 43 | 81 | 37 | 39 | 16 | **This study** |
| 66 | PG37089.J20 | 2014 | Late | PLASMA | **This study** | 26 | 30 | 42 | 72 | 37 | 29 | 13 | **This study** |
| 67 | PG37089.J83 | 2014 | Late | PLASMA | **This study** | 25 | 33 | 43 | 74 | 37 | 29 | 14 | **This study** |
| 68 | PG37091.J41 | 2014 | Late | PLASMA | **This study** | 33 | 26 | 43 | 69 | 37 | 33 | 10 | **This study** |
| 69 | PG37080.J6A | 2014 | Late | PLASMA | **This study** | 25 | 28 | 38 | 66 | 37 | 28 | 14 | Patil et al. *J Virol* (2016) 90(7):3446-57. |
| 70 | PG37080v1.J17 | 2014 | Late | PLASMA | **This study** | 25 | 28 | 38 | 66 | 37 | 28 | 14 | Patil et al. *J Virol* (2016) 90(7):3446-57. |
| 71 | PG37080.J158 | 2014 | Late | PLASMA | **This study** | 25 | 26 | 38 | 64 | 37 | 28 | 14 | Patil et al. *J Virol* (2016) 90(7):3446-57. |

*Note: Clinical samples collected post 6 months of documented infection and beyond are document as ‘late” in this study.*
