# Supplementary material for: Neutralization diversity of HIV-1 Indian subtype C envelopes obtained from cross sectional and followed up individuals against broadly neutralizing monoclonal antibodies having distinct gp120 specificities
Source: Retrovirology. 2021 May 14;18:12. doi: 10.1186/s12977-021-00556-2 (PMC8120817; doi:10.1186/s12977-021-00556-2)
Supplement: Supplementary file 2 — Additional file 2: Table S2. Mapping key amino acid substitutions associated with resistance to the bnAbs. [file 12977_2021_556_MOESM2_ESM.docx]

**Table S2. Key amino acid residues associated with neutralization resistance**

| **VRC01** | | **CAP256-VRC26.25** | | **PGDM1400** | | **PGT121** | |
| --- | --- | --- | --- | --- | --- | --- | --- |
| **Envelopes** | **Resistant signatures** | **Envelopes** | **Resistant signatures** | **Envelopes** | **Resistant signatures** | **Envelopes** | **Resistant signatures** |
| 5-4.J18 | 234NGT, 364H | VB51.J22 | 165I, R166K, V169T 332NIS | VB51.J22 | 165I, 332NIS | 2.J9 | 252K, 293E,332TIN, 334N, 336S, 448N |
| LT1_09.J8 | 234NGT | VB96.J21 | R166K, 332NIS | VB96.J21 | K130N, 160KAT, 332NIS | 2-3.J7 | R252K,N289,293E,N332T, S334N,A336S,N448 |
| NISA-N101.J12 | 234NGT | VB97.J10 | R166T, 332NIS |  |  | 2-3.J4 | R252K,N289,293E,N332T, S334N,A336S,N448 |
| PG37009v2.eJ9 | None found | VB97.J15 | R166T, 332NIS |  |  | 2-3.J17 | R252K,N289,293E,N332T, S334N,A336S,N448 |
| PG37112v2.J5 | 234NGT | VB106.J38 | V169Q,332NIS |  |  | 2-5.J3 | R252K,N289,293E,N332T, S334N,N448 |
| PG37112v2.J9 | 234NGT | 3-J16 | 165I, 332NIS |  |  | 2.5.J11 | R252K,N289,293E,N332T, S334N,N448 |
| PG37066.J1 | None found | 3-3.J9 | 165I, 332NIS | 3-3.J9 | 165I, 332NIS | 2-7.J1 | R252K,N289,293E,N332T, S334N,A336S,N448 |
| PG37081.J36 | 234NGT | 3-5.J25 | 165I, 332NIS | 3-5.J25 | 165I, 332NIS | 11.J25 | I165L,R252K,N289N,E293,H330Y,N332D,S334N,A336T |
|  | | 4-2.J41 | 165I | 4-2.J41 | I161V, 165I | 11.J28 | I165L,R252K,N289N,E293,H330Y,N332D,A336T |
|  |  | INDO SA NLR 29.J80 | 165I, V169E,332NIS | INDO SA NLR 29.J80 | 165I, V169E, 332NIS | INDO SA NLR 29.J80 | R252K, N289N |
|  |  | INDO SA NLR 29.J11 | 165I |  |  |  |  |
|  |  | PG37072.J12 | 165I | PG37072.J12 | 165I | PG37072.J12 | K155, R252K,N289,H330Y,N332Q |
|  |  | PG37087.J39 | R166K | PG37087.J39 | 160KTT | PG37087.J39 | I165L, R252K, N289, H330Y, N332N, S334N, N448 |
|  |  | PG37087.J44 | R166K | PG37087.J44 | None found | PG37087.J44 | 165L, 252K, 289NET, 330Y, 332NIN, 334N, 448N |
|  |  | PG37091.J41 | 165I,332NIS | PG37091.J41 |  | PG37091.J41 | 165I, 332NIS |
|  |  | PG37080.J6A | 165I,167G, 211E | PG37080.J6A | K130N, 165I, 167G, 211E | PG37080.J6A | K155R, R252K, N289N, E293, Q328K, N332T, S334N, N448 |
|  |  | PG37080v1.J17 | 165I,167G, 211E | PG37080v1.J17 | K130N, 165I, 167G, 211E | PG37080v1.J17 | K155R, R252K, N289N, E293, Q328K, H330Y, N332T, S334N, N448N |
|  | | | | VB52.J29 | K130N, 165I, 332NIS | PG37112v2.J9 | K155R,R252K,N289,E293,H330Y,N448 |
|  |  |  |  | VB95.J22 | 165I, 332NIT | PG37072.J12 | K155, R252K,N289,H330Y,N332Q |
|  |  |  |  | VB105.J10 | 332NIS | PG37072.J16 | R252K, N289N, E293, H330Y,N332Q |
|  |  |  |  | 2-7.J1 | None found | PG37066.J1 | R252K,E293,I307V,N448 |
|  |  |  |  | 11.J25 | None found | PG37087.J39 | I165L, R252K, N289, H330Y, N332N, S334N, N448 |
|  |  |  |  | 11.J28 | None found | PG37087.J44 | 165L, 252K, 289NET, 330Y, 332NIN, 334N, 448N |
|  |  |  |  | PG37009v2.eJ9 | K130N, 161I | PG37089.J17 | 252K, 289NES, 293E, 307V, 330Y, 448N |
|  |  |  |  | PG37112v2.J5 | K130N, 160KVT, I161V, 165I, V169Q, 332NIS | PG37080.J158 | R155K, R252K, N289N, E293, Q328K, H330Y, N332T, S334N, N448N |
|  |  |  |  | PG37112v2.J9 | K130N, 160KVT, I161V, 165I, V169Q, 332NIS |  | |
