# Supplementary figures and images for: Neutralization diversity of HIV-1 Indian subtype C envelopes obtained from cross sectional and followed up individuals against broadly neutralizing monoclonal antibodies having distinct gp120 specificities
Source: Retrovirology. 2021 May 14;18:12. doi: 10.1186/s12977-021-00556-2 (PMC8120817; doi:10.1186/s12977-021-00556-2)

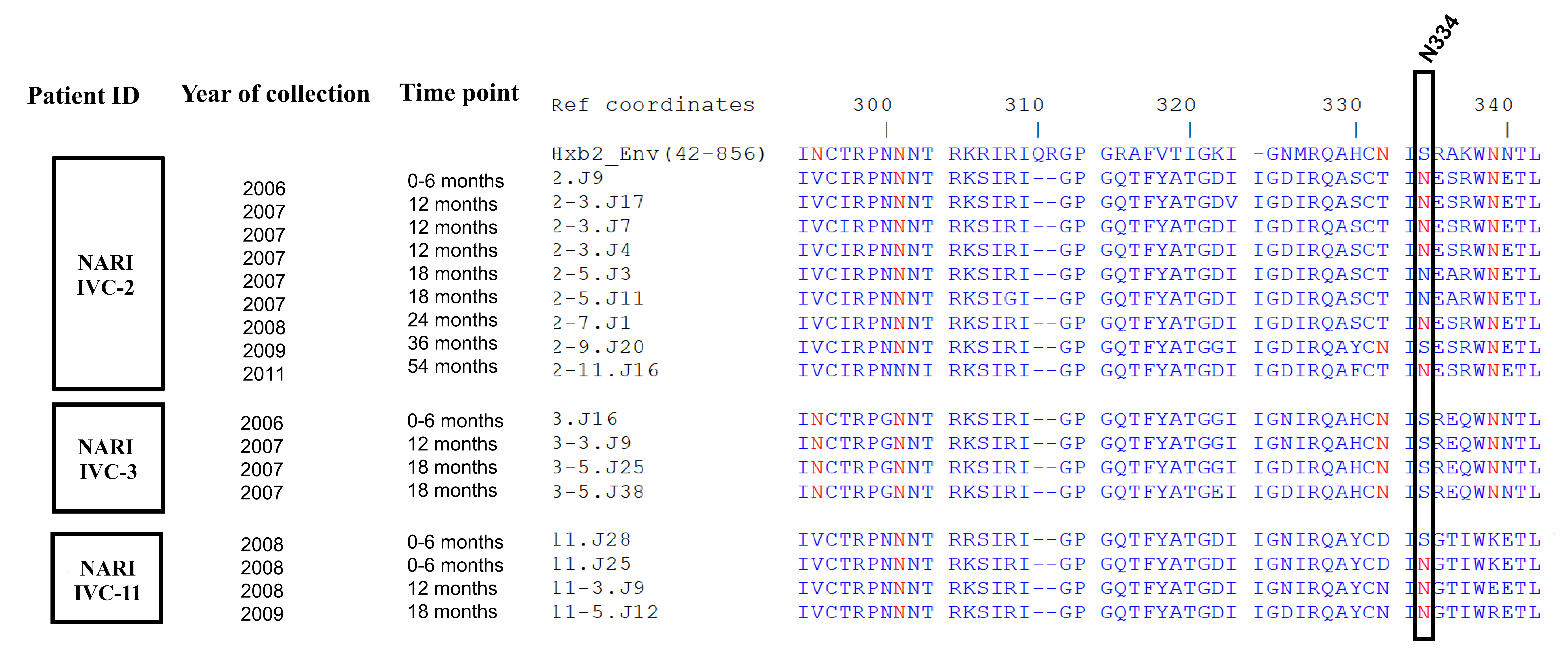

Supplement: Supplementary file 3 — Additional file 3: Figure S1. Amino acid sequence alignment of V3 region of the autologous HIV-1 env obtained from NARI IVC-2, NARI IVC-3 and NARI IVC-11 collected at different time points in a span of 2006–2011 to denote the frequency of occurrence of N334 glycosylation. [file 12977_2021_556_MOESM3_ESM.tif]
